# Electroencephalography Mu Rhythm Changes and Decreased Spasticity After Repetitive Peripheral Magnetic Stimulation in Patients Following Stroke

**DOI:** 10.3389/fneur.2020.546599

**Published:** 2020-09-29

**Authors:** Shugeng Chen, Yang Li, Xiaokang Shu, Chuankai Wang, Hewei Wang, Li Ding, Jie Jia

**Affiliations:** ^1^Department of Rehabilitation Medicine, Huashan Hospital, Fudan University, Shanghai, China; ^2^Department of Rehabilitation Medicine, Shanghai Jing'an District Central Hospital, Shanghai, China; ^3^School of Mechanical Engineering, Shanghai Jiaotong University, Shanghai, China; ^4^National Clinical Research Center for Aging and Medicine, Huashan Hospital, Fudan University, Shanghai, China

**Keywords:** event-related desynchronization, laterality, mu rhythm, spasticity, peripheral magnetic stimulation, stroke

## Abstract

**Background:** Spasticity is common among patients with stroke. Repetitive peripheral magnetic stimulation (rPMS) is a painless and noninvasive therapy that is a promising approach to reducing spasticity. However, the central mechanism of this therapy remains unclear. Changes in cortical activity and decreased spasticity after rPMS intervention require further exploration. The aim of this study was to explore the electroencephalography (EEG) mu rhythm change and decrease in spasticity after rPMS intervention in patients with stroke.

**Materials and methods:** A total of 32 patients with spasticity following stroke were recruited in this study and assigned to the rPMS group (*n* = 16) or sham group (*n* = 16). The modified Ashworth scale, modified Tardieu scale, and Fugl–Meyer assessment of the upper extremity were used to assess changes in upper limb spasticity and motor function. Before and after the rPMS intervention, EEG evaluation was performed to detect EEG mu rhythm changes in the brain.

**Results:** After one session of rPMS intervention, spasticity was reduced in elbow flexors (*p* < 0.05) and wrist flexors (*p* < 0.05). Upper limb motor function measured according to the Fugl–Meyer assessment was improved (*p* < 0.05). In the rPMS group, the power of event-related desynchronization decreased in the mu rhythm band (8–12 Hz) in the contralesional hemisphere (*p* < 0.05).

**Conclusions:** The results indicate that rPMS intervention reduced spasticity. Cortical activity changes may suggest this favorable change in terms of its neurological effects on the central nervous system.

## Introduction

Spasticity develops mainly as a result of disruption in the balance of supraspinal inhibitory and excitatory inputs to the spinal cord after the formation of a central lesion (1). Lance et al. attributed spasticity to the hyperexcitability of the stretch reflex in upper motor neuron syndrome (2). Subsequently, Pandyan et al. identified spasticity as arising from the involuntary activation of muscles by upper motor neurons (3). Moreover, Málly et al. reported that spasticity following stroke could be modified through stimulation of the ipsilesional or contralesional hemisphere (4). Activity in these hemispheres may influence spasticity, and cortical activity may change with changes in spasticity. Exploring the cortical activity changes during interventions in reducing spasticity can facilitate improvements in the assessment and treatment of spasticity.

Various approaches have been adopted in the treatment of spasticity following stroke, including botulinum toxin injection (BTX), stretching, orthosis, neuromuscular electrical stimulation (NMES), and magnetic stimulation. BTX, which is a pharmacological method, was reported to be effective (5). However, this treatment is expensive and invasive. Stretching and orthosis are routinely used in rehabilitation, but only weak evidence supports their effects on spasticity (6, 7). Studies have demonstrated that NMES and repetitive transcranial magnetic stimulation (rTMS) can reduce spasticity and increase upper limb motor function in patients following stroke (8, 9), but evidences supporting their efficacy are limited. Repetitive peripheral magnetic stimulation (rPMS) is a noninvasive and painless treatment that is applied to the peripheral nerve systems and peripheral limbs. In addition to electrically stimulating the targeted nerves, this treatment generates more proprioceptive sensory input than NMES does through its magnetic field (10). Therefore, the stimulation effects of rPMS are stronger than those of NMES.

Numerous studies have investigated the effects of rPMS on motor rehabilitation, with a particular focus on spasticity. Both single session (11) and multiple sessions (12) of rPMS was reported to significantly reduce spasticity in patients with central paresis. Their results revealed improved clinical outcomes for the treatment of spasticity and identified positive effects of rPMS on the central nervous system. Struppler et al. was the first to report that rPMS could induce sensorimotor integration and improve cognitive ability and subsequently demonstrated that effects on the frontoparietal network could be enhanced through rPMS during a single session (13, 14). Gallasch et al. used functional magnetic resonance imaging (fMRI) to reveal that rPMS caused a short-lasting modulation of the sensorimotor cortex (15). Another study reported that the central nervous system was modulated by both external and internal factors that modulate sensor perception and motor movement (16). Changes in cortical activity following rPMS may be due to its neurological modulating effects.

By collecting electroencephalography (EEG) signals from the scalp, Comani et al. detected real-time cortical electrical activity and identified instant and synchronized changes in the cortex (17). To date, several studies have reported relationships between EEG features and spasticity. Stronger EEG mu rhythm desynchronization in the ipsilesional hemisphere was reported to be associated with higher spasticity in a motor imagery task and decreased spasticity was associated with activation of EEG over the ipsilesional sensorimotor network after a robot-assisted bilateral arm training (18, 19). Both positron emission tomography (PET) and fMRI have also been used to assess changes in cortical activity after rPMS (13, 15). Meanwhile, the EEG-fMRI technology can be a new method for post-stroke assessment (20). However, EEG has not yet been applied to this task. These results suggest that EEG could be used to evaluate the neurological modulating effects of rPMS on spasticity. Additionally, event-related synchronization (ERS) and desynchronization (ERD), which are increases and decreases in the EEG power band, respectively, indicate changes in cortical activity (21). Mu rhythm ERD can reflect motor cortex activity during motor execution and observation (22, 23) and is a reliable indicator for and electrophysiological correlate of motor cortex activation (24, 25). Thus, mu rhythm ERD could be used to detect cortical activity in patients with spasticity following stroke and identify changes after rPMS treatment.

The cortical activity changes along with spasticity decrease before and after rPMS intervention has not be investigated using EEG. The aim of the present study was to explore this possible central nerve effect. We used ERD power change to detect cortical activity changes after rPMS intervention. We hypothesized that the mu rhythm ERD power would decrease following a decrease in spasticity.

## Materials and Methods

### Research Subjects

**Figure 1:**
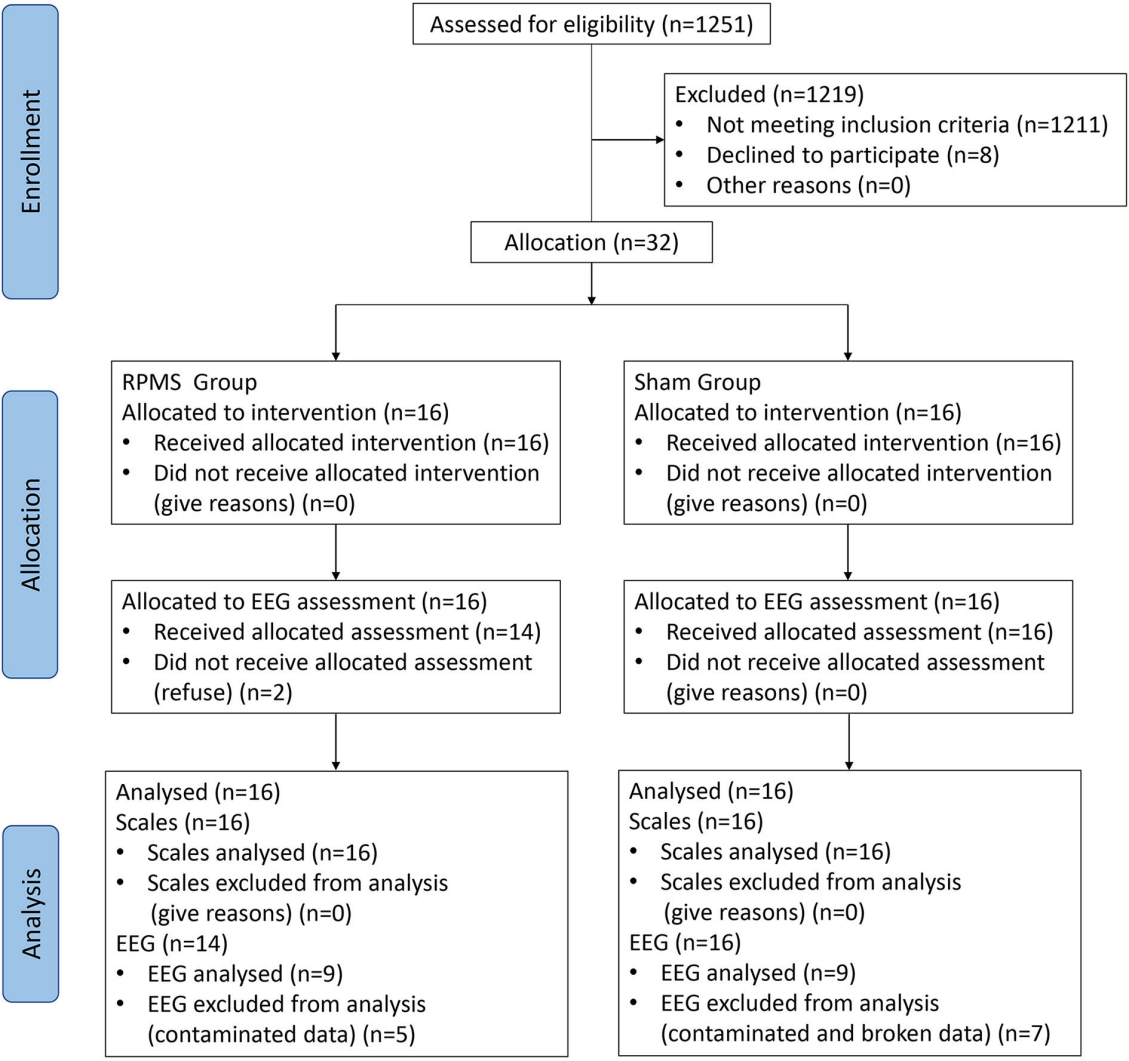
Flow chart of the study sample and procedures of the rPMS and sham groups.

Patients in the subacute or chronic stage of stroke were recruited from the Department of Rehabilitation Medicine, Huashan Hospital. The inclusion criteria for patients following stroke were (1) ischemic or hemorrhagic stroke diagnosed through computed tomography or MRI; (2) age in the range of 18–80 years; (3) at least 2 weeks since stroke onset; (4) spasticity (MAS score of a muscle in the upper limb or hand ≥ 1); and (5) ability to sit on a chair independently for at least 1 h. The exclusion criteria were (1) use of a cardiac pacemaker; (2) pregnancy; (3) allergy to EEG electrode cream; (4) any osteoarthrosis (including joint deformity) that could cause joint contracture in the hand or upper limb; and (5) unstable fracture in the paretic upper limb. A total of 32 patients met these criteria and were enrolled in this double-blinded study. Patients were allocated nonrandomly to the rPMS group (*n* = 16) or the sham group (*n* = 16). An independent researcher, who was blinded to the study allocation, conducted all assessments. This study was approved by the ethical committee of Huashan Hospital. All the patients signed informed consent forms in accordance with the Declaration of Helsinki. Figure 1 presents a flow diagram for the study. Table 1 lists the patients' demographic and baseline clinical characteristics.

**Table 1:** Demographic and baseline clinical characteristics of the patients.

** Patients**	**RPMS group**	**Sham group**	***p***
*n* = 32	16	16	–
Gender, *n* (%)			0.433
Male	10 (62.5)	13 (81.25)	–
Female	6 (37.5)	3 (18.75)	–
Age (years)	49.0 ± 18.2	45.6 ± 8.3	0.5
Type of injury			0.054
Ischemia	8 (50)	2 (12.5)	–
Hemorrhage	8 (50)	14 (87.5)	–
Affected limb			0.716
Left	7 (43.75)	5 (31.25)	–
Right	9 (56.25)	11 (68.75)	–
Time post-stroke (month)	37.4 ± 42.0	22.8 ± 26.7	0.25
FMA-UE (total score)	29.1 ± 10.0	22.1 ± 10.2	0.06
MAS (total score)	6.3 ± 2.3	4.9 ± 1.3	0.051

*FMA-UE, Fugl–Meyer assessment of the upper extremity; MAS, modified Ashworth scale*.

### Protocol for rPMS Intervention

**Figure 2:**
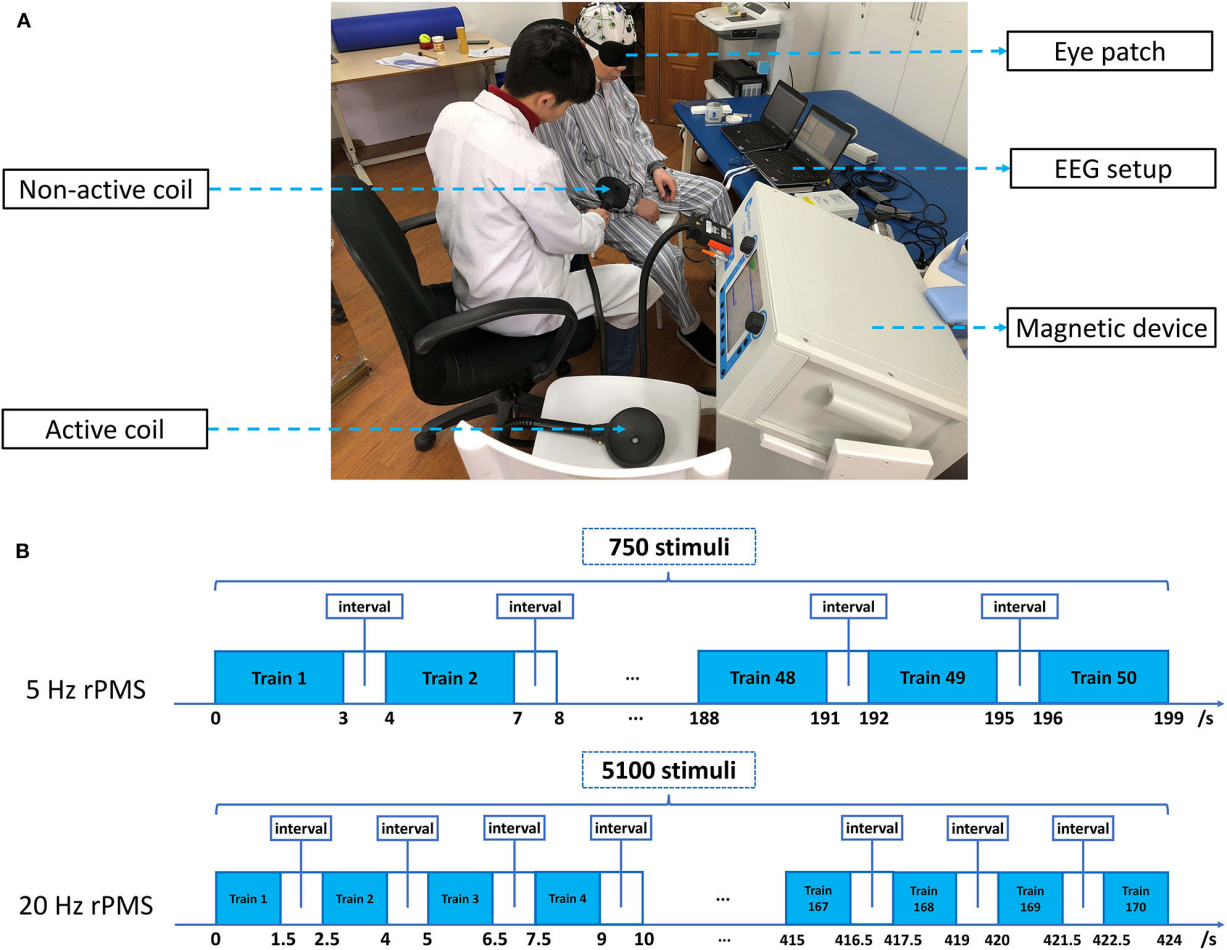
Experimental setup and intervention protocol. **(A)** Experimental setup for the sham group. The patient was sitting in the chair wearing an eye patch and was treated with a nonactive coil; the active coil made noises according to its normal operation as in the rPMS group. **(B)** Intervention protocol and the treatment block. The 5-Hz rPMS intervention comprised 50 trains of 3 s, and the 20-Hz rPMS intervention comprised 170 trains of 1.5 s. A 1-s interval was applied between each train.

Figure 2A shows the experimental setup for the sham group. We attached a second coil to the patients' upper limbs in the sham group with a real coil operated nearby on a chair to produce a simulated voice. The patients were asked to wear an eye patch and sit in the chair [the sitting posture was adopted from a previous study (26)] during the rPMS or sham rPMS intervention. The therapist arranged the patients' upper limbs in a suitable position with one hand and applied magnetic stimulation with the other hand. The parameters in the sham group were all the same as those in the rPMS group.

A Mag-Pro R30 magnetic device (Tonica Elektronik A/S, Denmark) and an MMC-140 parabola coil (Tonica Elektronik A/S) were employed for stimulation. The coil was focused on stimulation and featured a parabola-shaped design. It had an inner diameter of 25 mm and external diameter of 120 mm, with a pulse width of 280 μs (Biphasic waveform). The power of this parabola-shaped design coil was more focused and stronger, which was suitable for peripheral stimulation. The intensity of stimulation was set to 100% of the muscle contraction threshold at a resting state and was adjusted for different muscles by the therapist. A single session of rPMS or sham rPMS was applied to the patients' upper limbs. The targeted muscle groups were shoulder adductors and extensors, elbow extensors and flexors, and wrist extensors and flexors. For muscle groups with spasticity, the therapy was applied at a low frequency of 5 Hz, with a high frequency of 20 Hz adopted for their antagonistic muscles. However, if the MAS score of the antagonistic muscle was ≥1, a 5-Hz stimulation was still used. Magnetic stimulation at 5 Hz with 15 stimulus per train was applied for a total of 750 stimuli, and magnetic stimulation at 20 Hz with 30 stimulus per train was applied for a total of 5100 stimuli. Trains were applied at 1-s intervals. The stimulation order was from the shoulder joint (proximal joint) to the wrist joint (distal joint), and the average duration of rPMS was ~30 min. The targeted points in the muscle groups were all started from shoulder adductors to extensors, from elbow flexors to extensors, and from wrist flexors to extensors. Stimulations were all started from the proximal point of the stimulated muscle to its distal point. Figure 2B illustrates the intervention protocol and the treatment block. The magnetic interventions were performed after the first EEG evaluation and before the second clinical measurement.

### Outcome Measurements

An experienced therapist who was blinded to the study allocation performed all the clinical measurements. The MAS and modified Tardieu scale (MTS) were used to assess spasticity in the shoulder, elbow, and wrist, which included six muscle groups, namely shoulder adductors and extensors, elbow extensors and flexors, and wrist extensors and flexors. MAS had possible scores of 0, 1, 1+, 2, 3, and 4, where 0 indicates normal muscular tone and 4 indicates fixed muscle contracture (27). In the MTS, R1 represents the angle of movement in a high velocity stretch, and R2 represents the passive range of movement in a low-velocity stretch. The Y value is obtained from the difference between R1 and R2. This can indicate the neurological component in spasticity among patients following stroke. The Fugl–Meyer assessment of the upper extremity (FMA-UE) was used to assess the motor function of the upper limb. It has a total of 66 points and mainly four subsections, including coordination and speed assessments of the proximal upper limb, wrist, and hand. These three scales (MAS, MTS, and FMA-UE) were applied before and immediately after the rPMS or sham intervention.

Questionnaires were completed by patients and their therapists. All patients were asked to report pain status in the affected upper limbs before, immediately after, and 24 h after the interventions. Before the patients' regular treatments the day after the intervention, therapists were asked if they noticed any changes in spasticity.

### EEG Evaluation

EEG evaluations were performed before and after the TMS interventions. We used 32 active Ag/AgCl electrodes (actiCAP, Brain Products, Gilching, Germany) to record EEG signals with a BrainAmp amplifier (Brain Products). The ground channel and reference channel were placed on the forehead and the right mastoid process, respectively. The electrode impedances were set to <5 kΩ. The band pass filter was set from 0 to 100 Hz, with a sampling rate of 200 Hz. Power line interference was minimized using a 50-Hz notch filter. The placement of all electrodes was based on the extended international 10–20 system (28).

**Figure 3:**
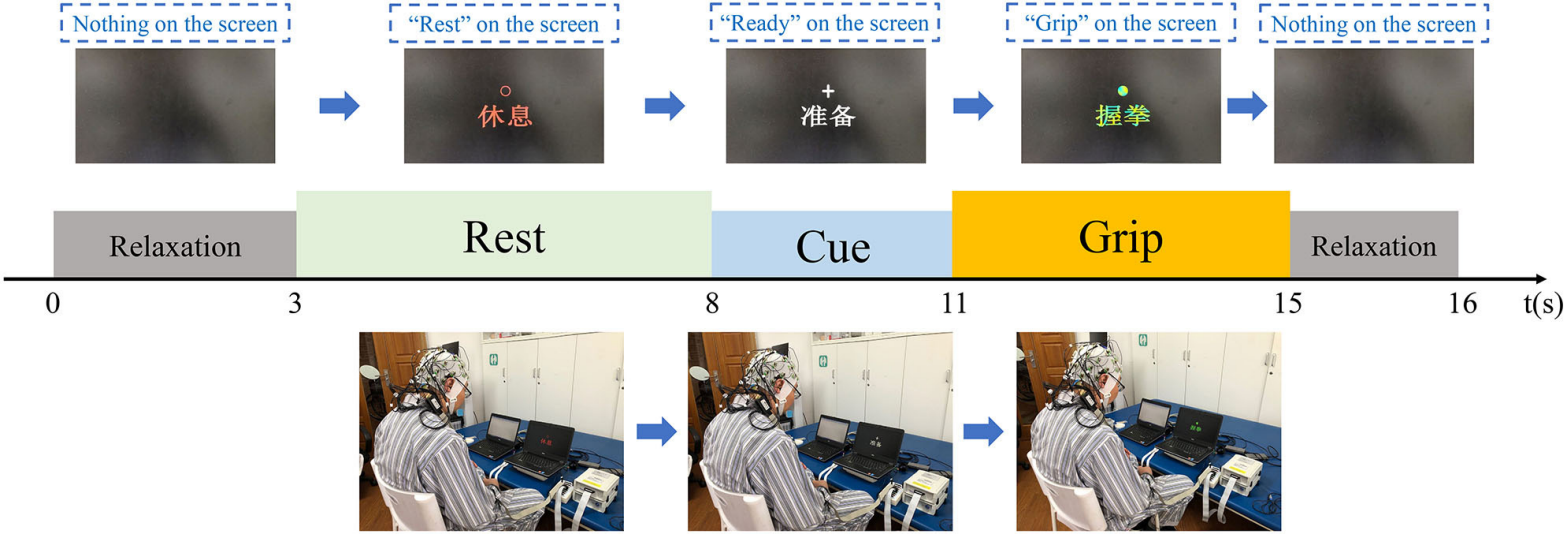
EEG evaluation paradigms. Each trial included a rest section, a ready section, a task section, and two relaxation sections.

During measurements, the patients were seated in a chair with their hands on their thighs in front of a computer. They were asked to look at the center of the computer screen and follow the cues on screen. Each evaluation comprised two blocks with 30 trials each, comprising a total of 60 trials. As shown in Figure 3, each trial lasted for 16 s and included relaxation with a black screen for 3 s, rest with no task for 5 s, a cue for preparation and concentration lasting 3 s, a grip task for the affected hand lasting 4 s, and further relaxation with a black screen for 1 s.

During both of the relaxation sections, the screen was black. In the rest section, the screen displayed a “rest” sign, and the patients were not required to do or think anything. During the cue section, a “ready” sign was displayed on the screen, and the patients were required to pay attention and prepare for the motor task, but they were required not to move during this section. Subsequently, a “grip” sign was displayed, and the patients were asked to attempt grip movements during for 4 s [the same design as that used in previous studies (29, 30)]. Finally, the patients were provided with a further relaxation section. The EEG data were collected during all five sections of the trials.

### EEG Processing

After recording, band-pass filter ranging from 1 to 50 Hz and average reference were applied during offline data analysis. The raw EEG data were preprocessed through independent component analysis (ICA) decomposition by using the EEGLAB toolbox V.11 software (http://sccn.ucsd.edu/eeglab/). ICA components, which represented ocular and muscular artifacts, were then manually removed from the data. The offline rejection automatically rejected artifacts exceeding 100 μV.

#### Time–Frequency Power Analysis

Event-related spectral perturbations (ERSPs) were first calculated by using Fast Fourier transformation. A three-cycle wavelet Hanning-tapered window was used to obtain a continuous measurement of the amplitude of a frequency component (31). Wavelet-transformed ERSP epochs were then computed for each stimulus requirement at each time points and each wavelet frequencies in order to form time–frequency plots. The color of each image pixel illustrates the latency time relative to the time-locking event as well as the amplification or attenuation at a given frequency. Following ERSP analysis, the absolute power was computed using Equation (1):

(1) $$\begin{array}{l} ERSP\text{\_}abs=10.ˆ\left(\frac{ersp\left\{1\right\}}{10}\right) \end{array}$$

where *ERSP_abs* is the difference in the outcome log spectrum from the baseline (32).

#### ERD Power Change Calculation

Change in ERD power was calculated using the method described by Pfurtscheller and Aranibar (24). For channels C3 and C4, it was calculated according to the average absolute power. The power values were obtained through ERSP analysis for the range of frequencies. Time–frequency plots were drawn using time windows from −4 to 7 s. The baseline period was from −3.5 to −2.5 s before the cue onset. The frequency band of interest was the mu rhythm (8–12 Hz) frequency band (33). We adopted channels C3 and C4 for further analysis because motor execution is reportedly associated with significantly stronger mu rhythm ERDs in C3 and C4 than in other channels (34).

### Statistical Analysis

We performed three-way repeated measures analysis of variance (ANOVA) with the within-subject factor of time (i.e., before and after therapy), various subsections (i.e., seven levels of the MAS, six levels of the MTS, and three levels of the FMA-UE), and the between-subject factor of group (i.e., therapy and sham). Two-way repeated measures ANOVA was also performed for the MAS, the MTS, and the FMA-UE, with time as the within-subject factor (i.e., before and after therapy) and group as the between-subject factor (i.e., therapy and sham). If a significant interaction was identified through two-way repeated measures ANOVA, then a paired *t*-test was adopted for *post hoc* analysis to compare MAS score, MTS score, and FMA-UE score before and after rPMS intervention. A *t*-test was conducted to compare the effects between groups.

Chi-square test (Fisher's Exact Test) was performed to test the difference in locations of cortical injury (subcortical, cortical or both subcortical and cortical injury) between groups. To assess the ERD power change of the electrodes between groups before and after treatment, three-way repeated measures ANOVA was performed to determine the minimum ERD, with time (i.e., before and after therapy) and electrode (i.e., C3 and C4) as the within-subject factors and group as the between-subject factor (i.e., therapy and sham). If a significant interaction was identified, then two-way repeated measures ANOVA was performed for the ERD power change. If the significant interaction was retained in two-way repeated measures ANOVA, then a paired *t*-test was applied for *post hoc* analysis to compare the ERD power change before and after rPMS intervention.

Analyses were conducted using SPSS version 23.0 (IBM Inc., Chicago, IL, USA). Continuous variables are presented as the mean ± standard deviation. An MAS score of +1 was converted into a score of 1.5, as described by Kim et al. (35). A *p*-value of <0.05 (two-sided) was considered to indicate a significant result. Bonferroni's correction was applied in *post hoc* multiple comparisons (α = 0.05/7 = 0.0071 for the MAS, α = 0.05/6 = 0.0083 for the MTS, and α = 0.05/3 = 0.0167 for the FMA-UE).

## Results

### Clinical Outcomes

Patients in the rPMS group (*n* = 16) were age- and sex-matched with patients in the sham group (*n* = 16; Table 1). The treatments in the rPMS and sham groups were completed without difficulty.

#### MAS

**Figure 4:**
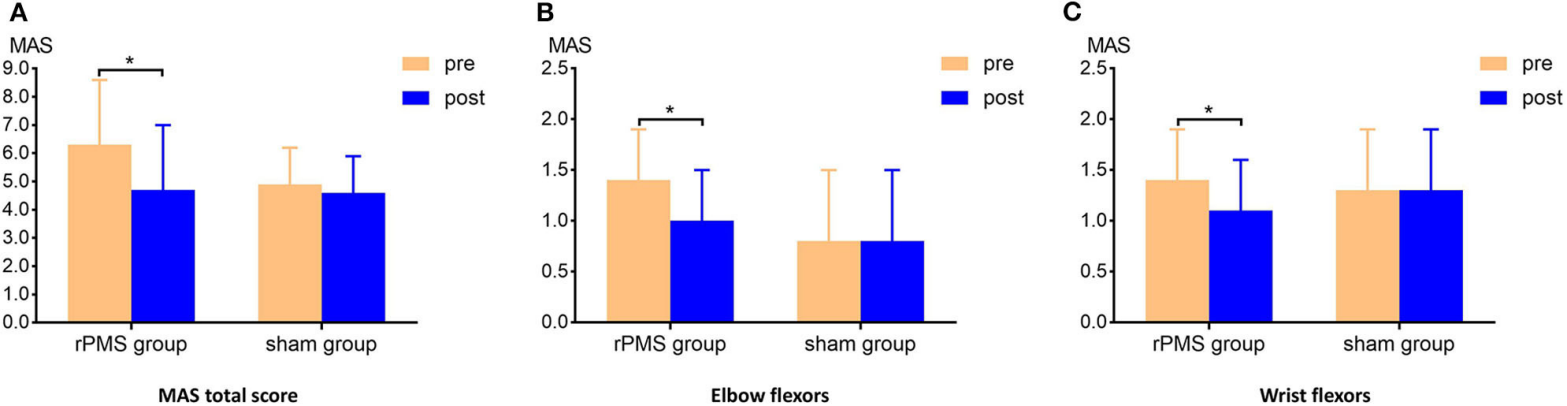
Clinical measurement of the MAS scores. **(A)** Comparison of the MAS total scores of the groups before and after the intervention. The MAS total score in the rPMS group differed significantly before and after the intervention; no difference was observed in the sham group. **(B,C)** Comparison of the MAS scores for the elbow flexors and wrist flexors for the groups before and after the interventions. Significant decreases were observed in the rPMS group, whereas no difference was noted in the sham group. *p < 0.0071, Bonferroni's correction.

Three-way repeated measures ANOVA results for the MAS scores revealed a significant interaction among muscle, time, and group [F_(2.562, 38.429)_ = 7.214, *p* = 0.001]. Table 2 lists the interaction results for time and group, with the two-way repeated measures ANOVA results for the MAS score of each muscle group and the total score. Further analysis revealed the MAS total score was significantly lower after the intervention in the rPMS group (*p* < 0.0071; Bonferroni's correction). In the rPMS group, the MAS scores for the elbow flexors decreased significantly from before therapy (1.4 ± 0.5) to after therapy (1.0 ± 0.5; *p* < 0.0071; Bonferroni's correction). The MAS scores of the wrist flexors also decreased significantly from before therapy (1.4 ± 0.5) to after therapy (1.1 ± 0.5; *p* < 0.0071; Bonferroni's correction). Figure 4 displays a bar chart of the MAS scores for the rPMS and sham groups. No significant difference was observed in the MAS scores before and after the intervention in the sham group.

**Table 2:** Comparisons of the MAS, MTS, and FMA-UE results in the rPMS and sham groups before and after rPMS or sham intervention.

**Subgroup**	**rPMS group (*****n*** **=** **16)**		**sham group (*****n*** **=** **16)**		**F**	***p***
	**Pre**	**Post**	**Pre**	**Post**		
**Muscle group (MAS)**						
Shoulder adductors	1.1 ± 0.8	0.8 ± 0.7	0.6 ± 0.6	0.6 ± 0.6	0.808	0.383
Shoulder extensors	1.1 ± 0.6	0.8 ± 0.7	1.0 ± 0.5	1.0 ± 0.5	2.882	0.110
Elbow extensors	0.8 ± 0.6	0.5 ± 0.6	1.0 ± 0.8	0.9 ± 0.8	2.872	0.111
Elbow flexors	1.4 ± 0.5	1.0 ± 0.5	0.8 ± 0.7	0.8 ± 0.7	9.000	0.009
Wrist extensors	0.6 ± 0.6	0.4 ± 0.6	0.2 ± 0.5	0.2 ± 0.4	0.678	0.423
Wrist flexors	1.4 ± 0.5	1.1 ± 0.5	1.3 ± 0.6	1.3 ± 0.6	5.952	0.028
Total score	6.3 ± 2.3	4.7 ± 2.3	4.9 ± 1.3	4.6 ± 1.3	11.766	0.004
**Passive motion (Y of MTS)**						
Shoulder abduction	33.3 ± 29.0	23.8 ± 21.4	18.4 ± 18.8	15.7 ± 17.6	1.735	0.198
Shoulder flexion	34.2 ± 30.9	25.5 ± 28.9	20.2 ± 16.1	17.9 ± 13.0	2.553	0.121
Elbow flexion	26.6 ± 28.4	12.8 ± 26.8	29.7 ± 37.5	29.8 ± 35.1	5.658	0.024
Elbow extension	64.4 ± 30.9	46.4 ± 33.4	57.3 ± 39.9	53.9 ± 41.3	9.589	0.004
Wrist flexion	22.0 ± 15.6	16.7 ± 13.3	16.2 ± 9.5	15.4 ± 9.6	2.526	0.122
Wrist extension	36.1 ± 14.6	19.8 ± 14.8	28.1 ± 29.0	29.0 ± 19.1	6.379	0.017
**FMA-UE**						
FMA (proximal)	20.0 ± 6.4	20.9 ± 6.3	16.0 ± 8.4	15.9 ± 8.3	8.571	0.010
FMA (wrist and hand)	4.9 ± 6.1	5.3 ± 6.4	2.2 ± 2.0	2.2 ± 2.0	5.870	0.022
FMA-UE (total score)	29.1 ± 10.0	30.4 ± 10.3	22.1 ± 10.2	22.1 ± 10.3	13.151	0.002

*pre, before rPMS or sham intervention; post, after rPMS or sham intervention; MAS, modified Ashworth scale; MTS, modified Tardieu scale; FMA-UE, Fugl–Meyer assessment of the upper extremity. The p-values represent interactions according to repeated measures ANOVA. All scores (MAS and FMA-UE) and degrees (Y values of MTS) are presented as the mean ± standard deviation*.

#### MTS

Three-way repeated measures ANOVA results for the MTS scores exhibited no significant interaction among motion, time, and group [F_(2.652, 39.779)_ = 0.732, *p* = 0.523]. However, a significant interaction was noted between time and group [F_(1, 15)_ = 14.472, *p* = 0.002]. Table 2 lists the interaction results for time and group, with two-way repeated measures ANOVA results for the Y values of the MTS of each motion. The results of further analysis demonstrated that the MTS scores of the elbow flexors in the rPMS group decreased significantly from before therapy (64.4 ± 30.9) to after therapy (46.4 ± 33.4; *p* < 0.0083; Bonferroni's correction). The MTS scores of the wrist flexors decreased from before therapy (36.1 ± 14.6) to after therapy (19.8 ± 14.8; *p* < 0.0083; Bonferroni's correction). No significant difference was observed in the MTS scores before and after the intervention in the sham group.

#### FMA-UE

Three-way repeated measures ANOVA results for the FMA-UE scores exhibited a significant interaction among subsection, time, and group [F_(1.292, 19.373)_ = 4.200, *p* = 0.045]. Table 2 lists the interaction results for time and group, with two-way repeated measures ANOVA results for FMA-UE in each subsection and the total score. The results of further analysis demonstrated that the baseline total FMA-UE scores in the rPMS and sham groups were similar (*p* > 0.05). After intervention, the total FMA-UE score in the rPMS group was significantly higher than that in the sham group (*p* < 0.0167; Bonferroni's correction). In the rPMS group, the proximal part of the FMA-UE score increased significantly from before therapy (20.0 ± 6.4) to after therapy (20.9 ± 6.3; *p* < 0.0167; Bonferroni's correction). No significant difference was observed in the total FMA-UE score or subsection scores before and after the intervention in the sham group.

#### Patient and Therapist Questionnaires

In the rPMS group, eight of the 16 patients (50%) reported pain in the upper limbs before the intervention; after the intervention, six patients reported pain relief and two reported a total absence of pain. For all of these patients, pain relief was maintained at 24 h. In the sham group, six of the 16 patients (37.5%) reported pain in the upper limbs before the intervention; after the intervention, all patients reported pain relief, but their pain returned the following day. The patients reported no other feelings of discomfort and considered the intervention to be helpful for them. All patients tolerated magnetic stimulation and the sham treatment.

According to the therapists responsible for the 32 patients, 14 of the 16 patients in the rPMS group (87.5%) and two of the 16 patients in the sham group (12.5%) exhibited a decrease in spasticity in the upper limbs during the day after treatment. The therapists were satisfied with the rPMS interventions.

### Change in Mu Rhythm ERD Power

In the compared EEG results, there were six subcortical stroke patients and three patients with both cortical and subcortical injury in the rPMS group. In the control group, there were seven subcortical stroke patients and two patients with both cortical and subcortical injury. There was no significant difference between groups (Fisher's Exact Test: *p* = 1.000).

#### Time–Frequency Plots for C3 and C4

**Figure 5:**
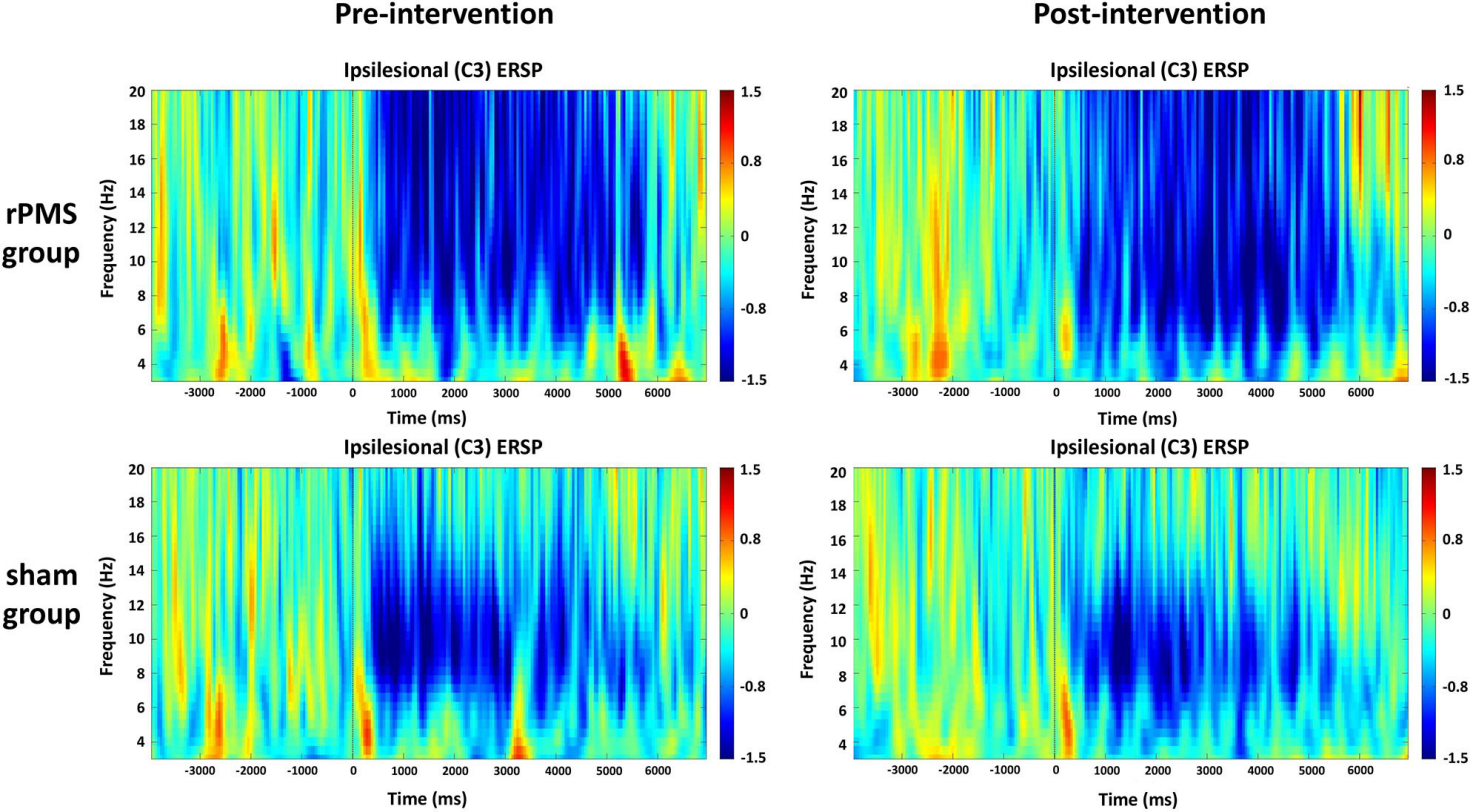
Average time–frequency plots for the ipsilesional hemisphere (C3) between the rPMS and sham groups.

**Figure 6:**
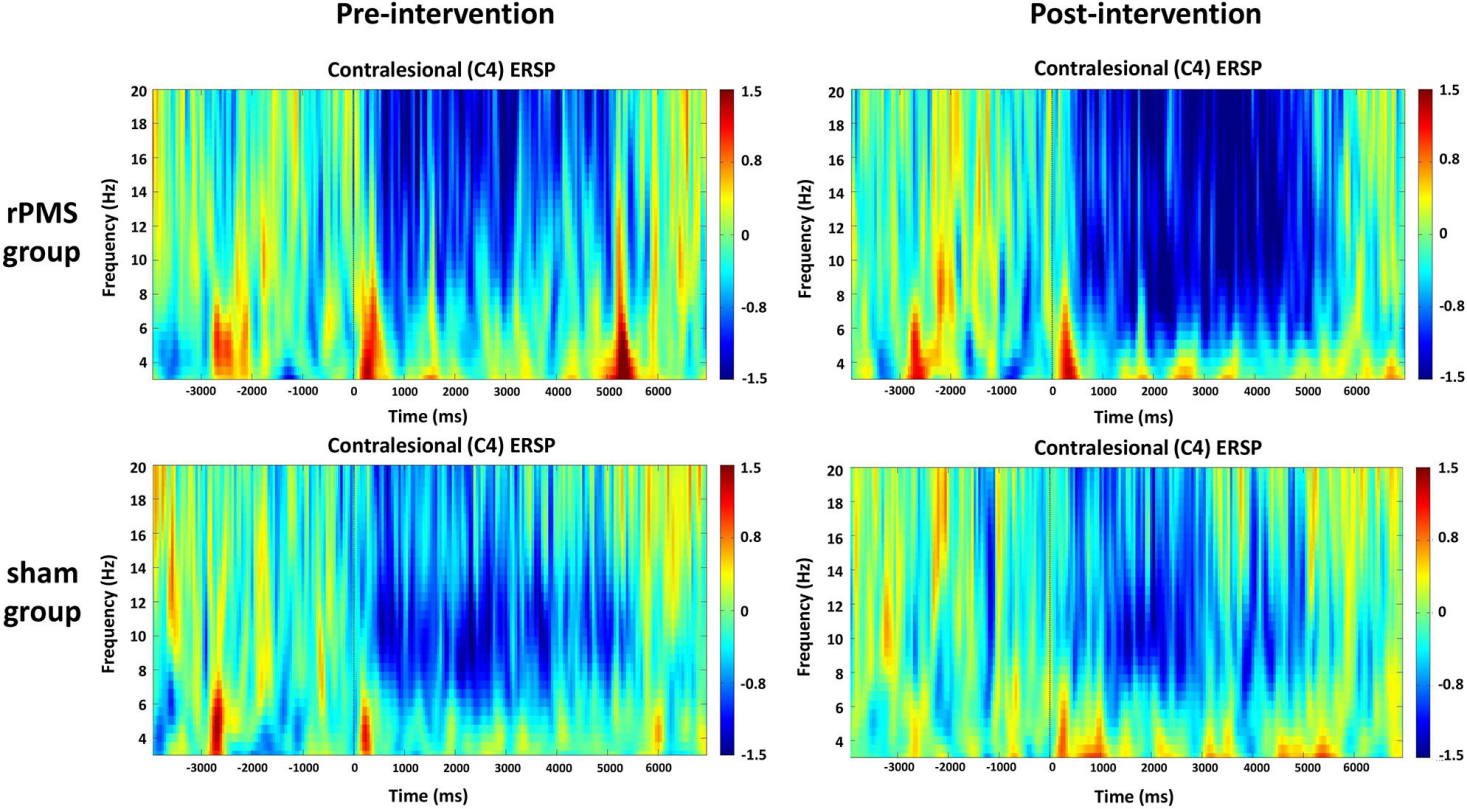
Average time–frequency plots for the contralesional hemisphere (C4) between the rPMS and sham groups.

The averaged ERSPs during the motor task onset (0 ms) in the rPMS group (*n* = 9) and sham group (*n* = 9) before and after the rPMS or sham intervention were exhibited in Figures 5, 6. A clear ERD (blue color) is present in both groups for the mu rhythm in the C3 and C4 channels between −4 and 7 s.

#### ERD Power Change

**Figure 7:**
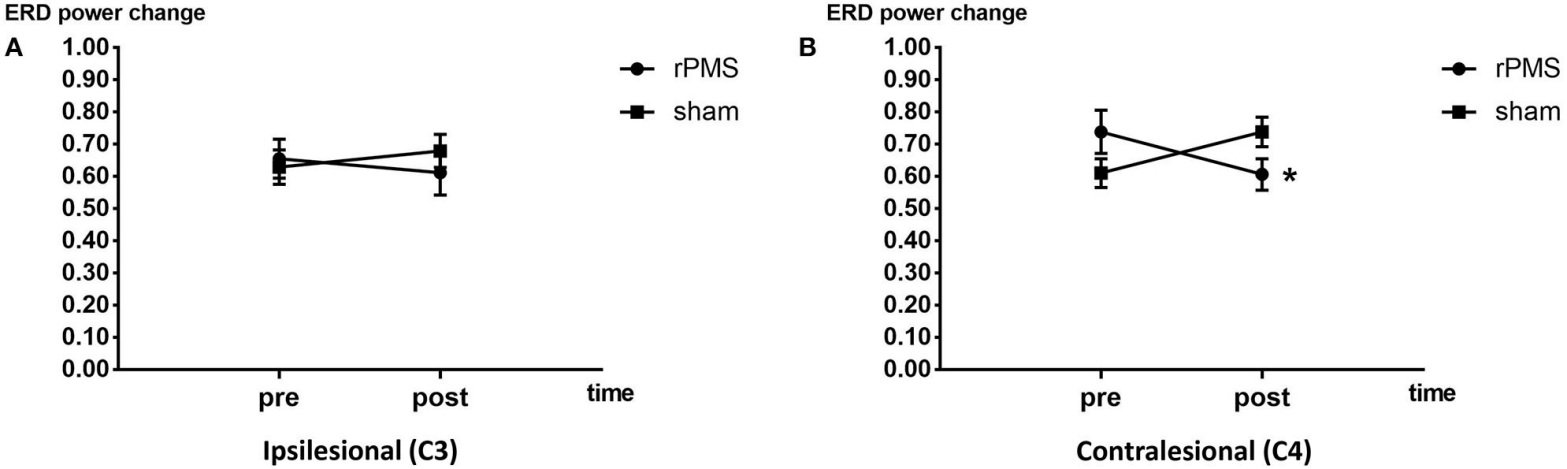
ERD power change plots of the rPMS and sham groups. **(A)** Difference in ERD power change before and after the rPMS or sham intervention in the rPMS and sham groups in the ipsilesional hemisphere (C3). **(B)** Difference in ERD power change before and after the rPMS or sham intervention in the rPMS (*p* < 0.05) and sham groups in the contralesional hemisphere (C4). *p < 0.05.

The ERD power changes of rPMS group (*n* = 9) and the sham group (*n* = 9) were showed in Figure 7. Three-way repeated measures ANOVA for the mu rhythm ERD power change revealed a significant interaction among electrode, time, and group [F_(1, 32)_ = 4.626, *p* = 0.047]. A follow-up two-way repeated measures ANOVA for ERD power change in each electrode revealed a significant interaction between time and group in channel C4 only [F_(1, 16)_ = 11.335, *p* = 0.04]. *Post hoc* analysis of channel C4 indicated a significant difference in ERD power change before and after the intervention in the rPMS group (*p* = 0.028); no significant difference was observed in the sham group.

## Discussion

In this study, a single session of rPMS or sham intervention was used to treat patients with spasticity following stroke. We explored changes in mu rhythm ERD before and after interventions. MAS and MTS scores were adopted to assess spasticity, and FMA-UE scores were used to assess motor function. Moreover, we calculated the ERD power change to detect cortical activity after the rPMS intervention, to explore the possible neurological mechanism of rPMS and spasticity decrease.

The changes in MAS and MTS scores indicated that spasticity immediately decreased in the elbow flexors and wrist flexors after a single session of rPMS. These results are consistent with the findings of Krewer et al., who reported a decrease in spasticity in the wrist flexors (measured using the MTS) after a single rPMS intervention session in patients following stroke (12). Their study also revealed a decrease in MTS scores for elbow extensors after multiple sessions of rPMS, but we observed a decrease in MTS scores for elbow flexors. Three possible explanations for this difference are detailed as follows. First, we applied rPMS to six muscle groups in the upper limb, namely the shoulder adductors and extensors, the elbow extensors and flexors, and the wrist extensors and flexors; however, Krewer et al. applied the treatment only to the elbow extensors and flexors and the wrist extensors and flexors. The additional stimulation of the shoulder joint in the present study may have influenced the elbow flexors. Second, we applied a single session of rPMS only, whereas Krewer et al. used multiple sessions of rPMS. The decrease in spasticity of the elbow extensors might have been obtained through the multiple sessions of rPMS. Finally, this difference may have been due to the difference in patients' baseline spasticity. However, in the present study, we adopted both the MAS and MTS to assess changes in spasticity; these scales produced consistent results, suggesting that the current results are reliable. The results of the present study are also in agreement with those of Werner et al., who identified a decrease in the wrist flexor spasticity (measured using the MAS) of patients with chronic cerebral injury (36). They used a single session of 5-Hz rPMS in combination with manual stretching, whereas stretching was not employed in the present study.

As stated by Patrick and Ada, utilizing the MAS to measure muscle tone is insufficient for determining the range of motion after restriction by neurological or mechanical components; however, this scale can provide a general understanding of changes in the spasticity of the upper extremity after application of rPMS (37). In the present study, all patients had an MAS score of <3 for all muscle groups in the upper limbs, which could have reduced the inter-subject variability. To further investigate the influence of this intervention on neural components, this study added MTS measurements. The MTS can provide more information on patients with spasticity than the MAS can (37, 38), and it enables a more sensitive evaluation of the neurological components of patients with spasticity because it can differentiate spasticity from contracture. Significant differences in Y value for the elbow and wrist flexors before and after rPMS suggest a neurological effect that was more evident than those of the soft tissue mechanical components. Struppler et al. reported a clear increase in the activation of the parieto-premotor network after rPMS treatment, and they also revealed positive modulation effects of rPMS on the cortex (13). Therefore, we explored changes in cortical activity following rPMS intervention.

This study revealed a significant reduction in spasticity following rPMS along with a stronger mu rhythm ERD in the contralesional hemisphere. This is consistent with the results of Pundik et al. (39) and Miyara et al. (40). Pundik et al. identified a positive correlation between decrease in spasticity and the strength of fMRI activation in the contralesional motor cortex. Miyara et al. reported that lower spasticity was correlated with stronger contralesional hemispheric activation following whole-body vibration through functional near-infrared spectroscopy (fNIRS). By contrast, the present study adopted EEG to measure cortical activity. Stronger activations in the contralesional hemisphere have been detected using fMRI (39) and fNIRS (40); this is in agreement with the EEG results of the present study, which revealed decreased ERD power in the contralesional hemisphere. This finding may indicate that activity changes in the contralesional hemisphere are associated with decreases in spasticity. Málly et al. also reported that spasticity can be modified by rTMS applied to the contralesional hemisphere (4). This further suggests that the contralesional hemisphere might be associated with spasticity changes.

In addition to the decrease in spasticity, motor function also exhibited improvement. The present study revealed a significant improvement in motor function in the upper limb, which was measured using the FMA-UE. However, Krewer et al. reported that a 20-min rPMS intervention had no significant effect on motor function according to the FMA-UE (12). In the present study, although increased scores were observed in 11 patients in the rPMS group (*n* = 16, 68.75%), only one patient had an improvement of 5 points—the minimum value for a clinically important difference (41). Therefore, the outcome of motor function improvements was consistent with the results of Krewer et al., suggesting that a single rPMS intervention session had limited effects on motor function recovery (12). Levin et al. (42) and Krewer et al. (12) have demonstrated that the prevalence of elbow extensor spasticity may affect motor function in the upper limbs. Thus, motor function could be improved by reducing spasticity. In this study, significant activations in the contralesional hemisphere were identified in the rPMS group. Although activations in the ipsilesional hemisphere were reported to promote motor recovery (43–45), activations in the contralesional hemisphere could also promote motor recovery among patients following severe stroke (46, 47). Further research with multiple sessions could be conducted to investigate the long-term effects in terms of motor recovery and changes in cortical activity.

The limitations of this study include the lack of a long-term intervention and another control group. We only applied a single rPMS intervention session to explore its short-term effects on patients. Its long-term effects on spasticity and functional recovery were not addressed. We also need another control group to clarify that the cortical activities change come from the pure rPMS intervention or the spasticity decrease as well.

## Conclusion

This study demonstrated that the mu rhythm ERD power decreased in the contralesional hemisphere following the decrease in spasticity after rPMS intervention. The results suggest that spasticity could be reduced by a single session of rPMS and that stronger mu rhythm ERD appears in the contralesional hemisphere after the intervention. The findings of this study support the suggestion that rPMS has neurological effects on spasticity, and providing a new approach to assessing and treating spasticity among patients following stroke.

## Data Availability Statement

The raw data supporting the conclusions of this article will be made available by the authors, without undue reservation.

## Ethics Statement

The studies involving human participants were reviewed and approved by the ethical committee of Huashan Hospital. The patients/participants provided their written informed consent to participate in this study. Written informed consent was obtained from the individual(s) for the publication of any potentially identifiable images or data included in this article.

## Author Contributions

SC and XS conceived and designed the study. YL and SC performed the intervention and the EEG evaluation. CW performed all clinical measurements. XS and SC organized the data. SC performed the statistical analyses and wrote the manuscript. HW, LD, and JJ reviewed and edited the manuscript. All authors contributed to the article and approved the submitted version.

## Conflict of Interest

The authors declare that the research was conducted in the absence of any commercial or financial relationships that could be construed as a potential conflict of interest.
